# Retinal vascular and structural recovery analysis by optical coherence tomography angiography after endoscopic decompression in sellar/parasellar tumors

**DOI:** 10.1038/s41598-023-40956-2

**Published:** 2023-09-01

**Authors:** Anil Ergen, Sebnem Kaya Ergen, Busra Gunduz, Sevgi Subasi, Melih Caklili, Burak Cabuk, Ihsan Anik, Savas Ceylan

**Affiliations:** 1 Department of Neurosurgery and Pituitary Research Center, School of Medicine, Kocaeli University, 41380 Kocaeli, Turkey; 2 Department of Ophthalmology, Kocaeli Seka State Hospital, Kocaeli, Turkey; 3 Department of Ophthalmology, School of Medicine, Kocaeli University, Kocaeli, Turkey

**Keywords:** Neurology, Oncology

## Abstract

We assessed the potential retinal microcirculation alterations for postoperative visual recovery in sellar/paraseller tumor patients with Optical Coherence Tomography Angiography (OCT-A). Two hundred ten eyes with sellar/parasellar tumor for which preoperative and postoperative (3 months) MRI Scans, Visual Acuity Test, Optical Coherence Tomography (OCT), OCT-A and, Visual Field Test data were available, besides 92 healthy eyes were evaluated. In the preoperative phase, significant reductions were observed in retinal vascular densities in various regions, including the Superficial Retinal Capillary Plexus (SRCP) (whole: *p* < 0.001, fovea: *p* = 0.025, parafovea: *p* < 0.001), Deep Retinal Capillary Plexus (DRCP) (whole: *p* < 0.001, fovea: *p* = 0.003, parafovea: *p* < 0.001), Peripapillary Vascular Density (PVD) (whole: *p* = 0.045, peripapillary: *p* < 0.001, nasal: *p* < 0.001, inferior: *p* < 0.001, temporal: *p* < 0.001), and Retinal Nerve Fiber Layer (RNFL) (nasal: *p* = 0.024, inferior: *p* < 0.001, temporal: *p* < 0.001, superior: *p* < 0.001) compared to the healthy control group. After surgery, the postoperative data of patients without chiasmal distortion were compared to their preoperative data. In the postoperative evaluation, significant increases were observed in vascular densities in patients without chiasmal distortion in the SRCP (whole: *p* < 0.001, parafovea: *p* = 0.045), DRCP (whole: *p* = 0.007, fovea: *p* = 0.006, parafovea: *p* = 0.040), PVD (peripapillary: *p* = 0.010, inferior: *p* < 0.001, temporal: *p* < 0.001, superior: *p* < 0.001), and RNFL (nasal: *p* = 0.011, inferior: *p* = 0.034, temporal: *p* = 0.046, superior: *p* = 0.011). Furthermore, significant associations were observed in the ROC analysis between the postoperative Visual Field Mean Deviation (VFMD) and SRCP (whole AUC = 0.793, *p* < 0.001, cut-off = 51.45, parafovea AUC = 0.820, *p* < 0.001, cut-off = 53.95), DRCP (whole AUC = 0.818, *p* < 0.001, cut-off = 55.95, parafovea AUC = 0.820, *p* < 0.001, cut-off = 59.05), PVD (temporal AUC = 0.692, *p* < 0.001, cut-off = 55.10), and RNFL (whole AUC = 0.690, *p* = 0.001, cut-off = 119.5, inferior AUC = 0.712, *p* < 0.001, cut-off = 144.75). These findings indicate a potential role of pre and post-operative OCT-A measurements in the assessment of surgical timing and postoperative visual recovery in patients with or without optic chiasm distortion.

## Introduction

Optic chiasm distortion by sellar/parasellar (SP) tumors with suprasellar extension may cause decreased visual acuity and visual field defect^1^. The postoperative recovery of vision loss by chiasmal distortion is one of the main factors affecting the quality of life^2^. With the endoscopic transsphenoidal approach, decompression of the optic apparatus can be achieved by tumor resection and a significant improvement can be observed in the visual field tests of the patients^3–5^.

Detection of visual loss due to optic chiasm distortion and visual recovery after decompression can be evaluated with visual field test (VFT) and Optical coherence tomography (OCT) for retinal nerve fiber layer (RNFL) thickness^6–8^. Apart from these tests, Optical Coherence Tomography Angiography (OCT-A) is a newly developed technique that provides quantitative data for noninvasively mapping the microcirculation of the retina and optic disc head in a short time^9^. This technology had become important to the evaluation of optic neuropathies and retinal vascular diseases^10^. This technique can also provide us information about the changes in retinal and optic nerve head vascularity caused by the distortion of the optic chiasm^11–15^.

In this study, we evaluated the changes in retinal parapapillary and perifoveal vessel density in patients with SP tumor. We also determined the relationship between OCT-A obtained data and other structural and functional tests such as visual field test (VFT), OCT (for Retinal Nerve Fiber Layer—RNFL parameters) and, MRI.

We researched to reveal that OCT-A is a useful tool for predicting visual recovery after the removal of pituitary tumors. The utility of OCT-A is determined in patients with optic chiasm distortion as well as without optic chiasm distortion on MRI. This study confirms that OCT-A features in pituitary tumors whether with or without optic nerve distortion have a value for determining optic neuropathy and prediction of postoperative visual recovery.

## Methods

This prospective observational study was conducted at Kocaeli University Pituitary Research Center*.* The study was performed in accordance with the ethical standards described in the Declaration of Helsinki and was approved by the local ethics committee of the Kocaeli University Faculty of Medicine, Kocaeli, Turkey *(GOKAEK-2022/8.12)*. Written informed consent was obtained from all patients.

### Research participants

One hundred five patients who operated endoscopically with the diagnosis of SP lesions from February 1, 2022 to October 1, 2022 in our clinic, participated in this study. All patients were operated by the same surgeon (Savas Ceylan) and total tumor mass resection was performed. The operation was performed by the endoscopic transsphenoidal approach which detailed before^16^.

Forty-six healthy subjects were recruited from staff and healthy volunteers who had undergone routine eye examinations. Written informed consent was obtained. None of these controls had a history of ophthalmologic or neurologic disease.

One hundred and five patients’ 210 eyes had detailed ophthalmologic examination preoperatively and 3 months postoperatively. Data including visual acuity, slit lamp bio microscopy, intraocular pressure (IOP) measurement, dilated fundus examination, standard automated perimetry, retinal nerve fiber layer (RNFL) thickness, and OCT-A examinations were collected from the patients. The presence of any other retinal or optic disc pathology on fundus examination or OCT imaging, history of systemic diseases such as diabetes, high blood pressure, rheumatologic or oncologic disease poor adaptation of ophthalmologic examination, were accepted as exclusion criterion. Intracranial surgery history or diagnosed intracranial lesions were excluded. The patients which had intrasellar limited microcystic lesions, MRI negative or < 5 mm adenomas, infrasellar located lesions (sphenoid sinus, clivus), and missing data were excluded. The patients which had complications such as postoperative sellar hematoma, recurrence, and residual tumor were also excluded.

The same ophthalmologist reviewed the results. Endocrinologic laboratory tests consulted by endocrinology. Pituitary MRIs were performed preoperatively, and postoperative first week and 3–4 months after the operation. The executive neurosurgeon evaluated the scans pre and postoperatively.

### Visual field test analysis

Visual field testing was performed with a Humphrey Field Analyzer using the 30-2 SITA-standard protocol (Humphrey 750I Visual Field Analyzer, Carl Zeiss Meditec, Dublin, CA, USA). Visual field tests were accepted as dependable when showing less than 20% fixation losses, and 33% false-positives, and false negatives rates. Mean deviation (MD) was used for the analysis.

### Optical coherence tomography angiography (OCT-A) measurements

The perifoveal and parapapillary microvasculature and the thickness of the retinal nerve fiber layer were analyzed using a Optovue AngioVue system based on decorrelation angiography (RTVue XR Avanti, Optovue Inc., Fremont, CA, USA) in all patients. This system includes a spectral domain OCT (SD-OCT) and AngioVue software enables the calculation of retinal thickness and vessel density in selected regions of the retina.

A 4.5 × 4.5 mm scan centered on the optic nerve head for evaluating the peripapillary region and a 6 × 6 mm scan centered on the fovea for evaluating the macular region were performed. Only high-quality scans with a scan quality index (SQI) > 7/10, without motion or blinking, were analyzed.

The software (version: 2018.0.0.14; Optovue, Inc) automatically calculated peripapillary vessel density and RNFL thickness. The measurement of the peripapillary vascular density (PVD) segment extending from the internal limiting membrane to the posterior boundary of the RNFL was analyzed in the whole scan area and all sections (superior, nasal, inferior, temporal).

The superficial and deep retinal capillary plexuses were automatically separated via layer segmentation with the software*.* The superficial retinal capillary plexus (SRCP) was comprised between the inner limiting membrane (ILM) and inner plexiform layer (IPL), while the deep retinal capillary plexus (DRCP) was comprised between IPL to outer plexiform layer (OPL). The software automatically calculated the vessel density in these two different retinal vascular networks.

### Brain imaging and optic chiasm distortion

All patients underwent 1.5 Tesla pituitary MRI with high-resolution coronal and sagittal T1-weighted contrast enhanced (T1 C+) sequences preoperative, postoperative first day, and postoperative third months. The clinical diagnosis of optic chiasm distortion was made based on magnetic resonance imaging (MRI) evidence of tumor distortion of the optic chiasm.

**Figure 1:**
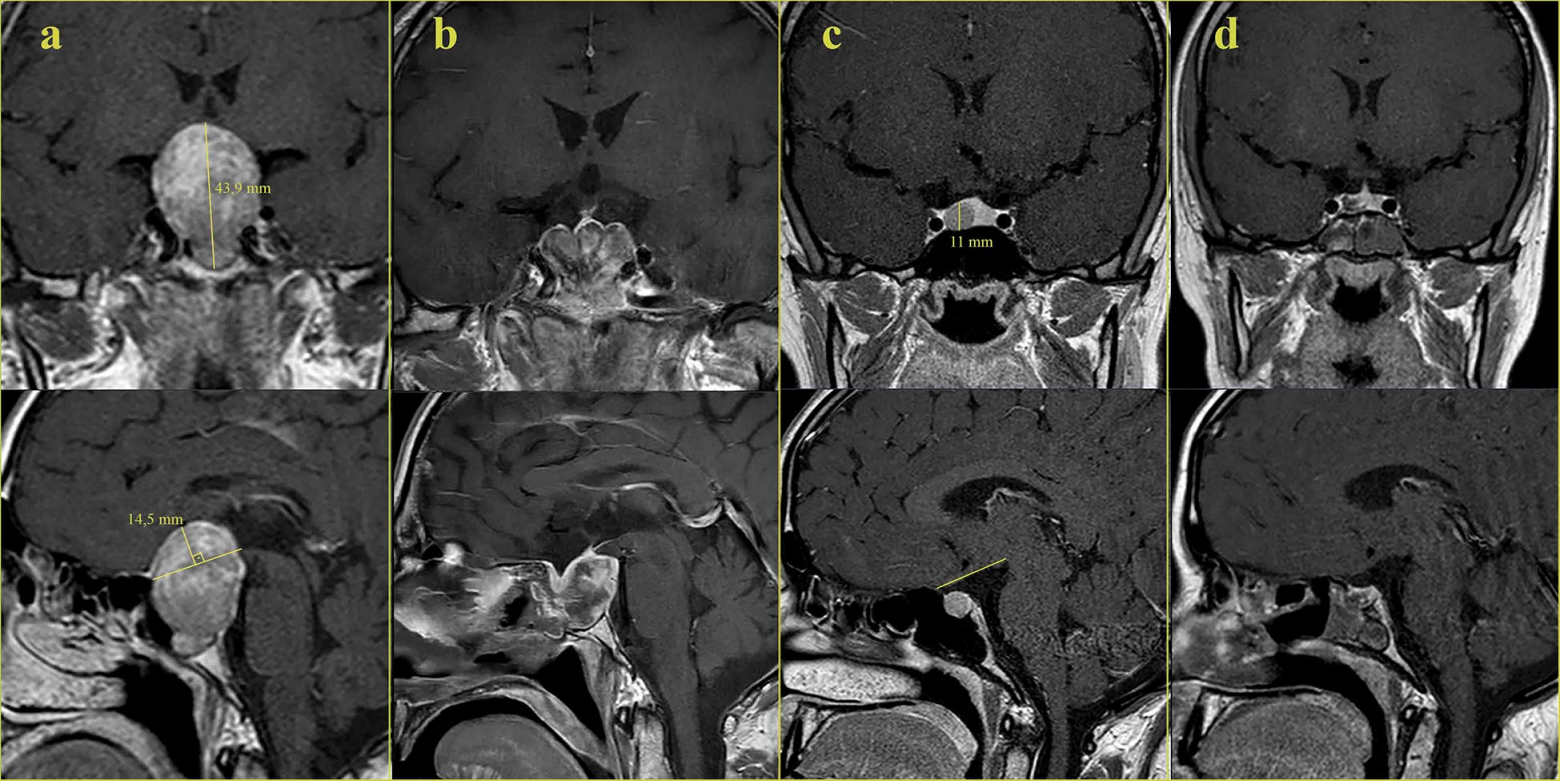
(**a**) Preoperative MRI views and tumor measurements of a patient with chiasmal distortion. (**b**) Postoperative MRI views of the same patient with chiasmal distortion. (**c**) Preoperative MRI views and tumor measurements of a patient without chiasmal distortion. (**d**) Postoperative MRI views of the same patient without chiasmal distortion.

Tumors' suprasellar extension was one of our reference criteria for visual deuteriation and recovery. The distance from the highest border of the lesion perpendicular to the sagittal reference line was defined as the suprasellar extension^17^. We modified the reference line according to the optic chiasm between the tuberculum sellae and the upper ventral edge of the Tectum, in the MRI midsagittal section (Fig. 1).

### Statistical analysis

Data analysis was performed using the Statistical Package for the Social Sciences (SPSS) 26.0 Statistics package program. The suitability of the numerical variables of the patients to the normal distribution was determined by looking at the skewness values. Parameters with normal distribution are presented as Mean ± S.D., while parameters that do not fit with normal distribution are presented as Mean ± S.D. Med. (Min.-Max.). Except for SRCP parafovea, PVD whole, PVD peripapillary, PVD nasal, PVD inferior, PVD temporal, and PVD superior values, it was observed to comply with the rules of normal distribution. The reference value taken in the normal distribution is between ± 1.5^18^. The chi-square test was used to compare the descriptive characteristics of the patients according to the presence of the tumor and the recovery status of the eyes. One Way ANOVA or Kruskal Wallis H Test was used to compare various preoperative parameters of the control group, patients with and without chiasmal distortion. Post Hoc Tests were applied for differences between groups. The Independent Sample T Test or Mann–Whitney U test was used to compare various preoperative parameters according to the recovery status of the eyes in patients with vision loss. Pearson or Spearman Correlation tests were used to examining the relationship between the recovery status of the eyes and the preoperative parameters. Correlation coefficient; A relationship between 0.00 and 0.30 was considered as a low-level, between 0.30 and 0.70 as a medium-level, and between 0.70 and 1.00 as a high-level relationship^19^. ROC analysis was performed to predict the possibility of recovery and non-recovery of the eyes from the preoperative parameters. In the whole study, the significance levels were carried out by considering the values of *p* < 0.05.

## Results

Three-hundred-two eyes of 151 participants were included in the study. There were 46 healthy control participants and 105 study subjects with SP tumors. A comparison of patients' demographic components according to the presence of SP tumor begins in Table 1.

**Table 1:** Comparison of demographic characteristics of patients.

Demographic characteristics		Patients’ eyes (n: 210)	
		Number	%
Year	< 40	104	49.5
	> 40	106	50.5
Sex	Female	136	64.8
	Male	74	35.2
Tumor nature	Secretory	104	49.5
	Non-secretory	106	50.5
Visual loss	None	61	29.0
	Recovery	104	49.5
	Nonrecovery	45	41.1
Tumor size	Micro	20	9.5
	Macro	161	76.6
	Giant	29	13.9
Optic chiasm relation	Distorted	128	60.9
	Undistorted	82	39.1
Year		41.64 ± 14.94 41 (8–72)	
		Med. ± S.S Med. (Min.–Max.)	

Pathological examination revealed the tumor distribution as 96 pituitary adenomas (60 non-secretory pitnets, 14 somatotropic pitnets, 9 corticotropic pitnets, 5 lactotrophic pitnets, 3 thyrotropic pitnets, 2 somatolactotropic pitnets, 3 gonadotropic pitnets), 4 craniopharyngiomas, 2 optic gliomas, 1 parasellar chordoma, 1 rathke cleft cyst, 1 tuberculum sellae meningioma.

Sixty-four of the patients had a variety of optic chiasm distortions caused by the tumor. The other 41 patients’ optic chiasm was undistorted by the tumor and in a normal proportion.

The comparison of the preoperative retinal parameters of the control group, patients with and without optic chiasm distortion is presented in Table 2.

**Table 2:** Preoperative parameters comparison of control group, patients with distortion and without distortion.

Parameters (preoperative)	Control^A^	Undistorted^B^	Distorted^C^	*p*	Difference
	Mean ± S.D	Mean ± S.D	Mean ± S.D		
SRCP whole^F^	52.53 ± 2.10	47.62 ± 4.09	46.41 ± 5.27	< 0.001**	**A > B > C**
SRCP fovea^F^	19.95 ± 6.80	19.29 ± 7.35	18.01 ± 8.46	0.025*	**A,B > C**
SRCP parafovea^K^	54.75 ± 3.04 55.20 (41.70–61.00)	48.35 ± 6.65 50.00 (30.8–57.20)	47.25 ± 8.63 49.40 (13.80–59.40)	< 0.001**	**A > B,C**
DRCP whole^F^	56.74 ± 6.55	49.54 ± 6.04 50.40 (32.60–60.60)	51.26 ± 7.10	< 0.001**	**A > B, C**
DRCP fovea^F^	38.21 ± 8.23	35.59 ± 7.00 35.5 (16.90–52)	34.13 ± 10.85	0.003**	**A,B > C**
DRCP parafovea^F^	59.38 ± 4.41	54.77 ± 4.82 55.90 (39.40–62.60)	54.89 ± 6.07	< 0.001**	**A > B, C**
FAZ^F^	302.35 ± 118.01	249.89 ± 131.65	289.59 ± 166.55	0.124	–
PVD whole^K^	49.98 ± 2.19 50.10 (44.10–56.60)	50.03 ± 2.31 50.00 (43.20–56.00)	46.91 ± 6.69 49.55 (30.20–55.40)	0.045*	**A, B > C**
PVD peripapillary^K^	52.41 ± 2.43 52.65 (44.20–58.80)	52.45 ± 2.68 52.05 (43.70–56.60)	47.85 ± 8.05 51.25 (27.60–58.30)	< 0.001**	**A, B > C**
PVD nasal^K^	49.57 ± 3.23 50.00 (41.00–58.00)	49.46 ± 3.51 49.75 (36.65–56.40)	44.49 ± 9.29 47.70 (20.85–58.60)	< 0.001**	**A, B > C**
PVD inferior^K^	53.78 ± 3.75 54.00 (45.00–63.00)	52.33 ± 4.21 52.60 (38.60–60.50)	48.74 ± 8.48 51.20 (0.00–61.25)	< 0.001**	**A, B > C**
PVD temporal^K^	54.53 ± 3.48 54.50 (42.00–64.00)	52.15 ± 2.80 52.50 (39.95–57.10)	49.23 ± 8.68 51.60 (0.00–61.25)	< 0.001**	**A > B,C**
PVD superior^K^	52.45 ± 3.15 53.00 (45.00–59.00)	51.59 ± 3.45 52.25 (43.05–58.75)	49.85 ± 8.45 52.03 (0.00–63.40)	0.341	–
RNFL whole^F^	115.28 ± 8.80	111.02 ± 18.72	108.57 ± 26.94	0.052	**A > C**
RNFL nasal^F^	102.97 ± 11.23	97.21 ± 22.73	94.61 ± 27.71	0.024*	**A > C**
RNFL inferior^F^	146.50 ± 15.7	130.99 ± 42.12	126.70 ± 46.15	0.001**	**A > B,C**
RNFL temporal^F^	75.97 ± 8.73	72.11 ± 33.27	71.66 ± 29.46	0.077	**A > B**
RNFL superior^F^	136.66 ± 13.28	124.66 ± 34.62	125.82 ± 38.00	0.017*	**A > B, C**

Significant values are in [bold].

*p < 0.05; **p < 0.01, F: One Way ANOVA Test, K: Kruskal Wallis H Test (Data presented with mean, median and standard deviation, minimum and maximum values).

There was no significant difference between the preoperative Foveal Avascular Zone (FAZ) (*p* = 0.124) and PVD superior (*p* = 0.34) values of the control group, with and without chiasmal distortion. There were significant differences between preoperative SRCP (whole *p* < 0.001, fovea *p* = 0.025, parafovea *p* < 0.001), DRCP (whole *p* < 0.001, fovea *p* = 0.003, parafovea *p* < 0.001), PVD (whole *p* = 0.045, peripapillary *p* < 0.001, nasal *p* < 0.001, inferior *p* < 0.001, temporal *p* < 0.001) RNFL (nasal *p* = 0.024, inferior *p* < 0.001, superior *p* < 0.001) values of those three groups.

The control group’s SRCP (whole *p* = 0.001, parafovea *p* = 0.001), DRCP parafovea (*p* = 0.001), PVD temporal (*p* < 0.001), RNFL (inferior *p* = 0.008, temporal *p* = 0.024, superior *p* = 0.013) parameters are significantly higher than patients without chiasmal distortion and SRCP (whole *p* < 0.001, fovea *p* = 0.009, parafovea *p* = 0.001), DRCP (whole *p* < 0.001, fovea *p* = 0.002, parafovea *p* < 0.001), PVD (whole *p* = 0.001, peripapillary *p* < 0.001, nasal *p* = 0.002, inferior *p* = 0.002, temporal *p* < 0.001) RNFL (whole *p* = 0.019, nasal *p* = 0.011, inferior *p* = 0.001, superior *p* = 0.012) parameters are significantly higher than patients with chiasmal distortion.

SRCP (whole *p* = 0.001, fovea *p* = 0.006), DRCP fovea (*p* = 0.006), PVD (whole *p* = 0.001, peripapillary *p* < 0.001, nasal *p* = 0.002, inferior *p* = 0.017 parameters of patients without chiasmal distortion, significantly higher than patients with chiasmal distortion.

The comparison of the preoperative and postoperative parameters of SP tumor patients without chiasmal distortion is shown in Table 3.

**Table 3:** Preoperative and postoperative retinal parameters comparison of patients without chiasmal distortion.

Parameters	Preoperative	Postoperative	*p*
	Mean ± S.D	Mean ± S.D	
VF MD^t^	− 1.79 ± 2.25	− 2.63 ± 2.66	< **0**.001**
SRCP whole^t^	47.62 ± 4.09	49.61 ± 3.40	< **0**.001**
SRCP fovea^t^	19.29 ± 7.35	20.85 ± 6.92	0.093
SRCP parafovea^z^	48.35 ± 6.65 50.00 (30.8–57.20)	49.40 ± 5.25 50.60 (32.60–61.50)	**0**.045*
DRCP whole^t^	49.54 ± 6.04 50.40 (32.60–60.60)	51.35 ± 6.46	**0**.007**
DRCP fovea^t^	35.59 ± 7.00 35.5 (16.90–52)	37.88 ± 7.15	**0**.006**
DRCP parafovea^t^	54.77 ± 4.82 55.90 (39.40–62.60)	55.50 ± 4.69	**0**.040*
FAZ^t^	249.89 ± 131.65	261.18 ± 106.36	0.184
PVD whole^z^	50.34 ± 2.65 50.60 (44.2–58.80)	50.03 ± 2.31 50.00 (43.20–56.00)	0.185
PVD peripapillary^z^	52.45 ± 2.68 52.05 (43.70–56.60)	53.44 ± 3.34 53.55 (45.70–62.70)	**0**.010**
PVD nasal^z^	49.46 ± 3.51 49.75 (36.65–56.40)	49.88 ± 3.97 50.50 (37–56.90)	0.118
PVD inferior^z^	52.33 ± 4.21 52.60 (38.60–60.50)	55.42 ± 4.14 55.20 (45.70–63.50)	< **0**.001**
PVD temporal^z^	52.15 ± 2.80 52.50 (39.95–57.10)	55.93 ± 3.10 56.20 (46.45–62.50)	< **0**.001**
PVD superior^z^	51.59 ± 3.45 52.25 (43.05–58.75)	54.00 ± 3.54 53.68 (44.90–62.00)	< **0**.001**
RNFL whole^t^	111.02 ± 18.72	113.07 ± 19.27	0.155
RNFL nasal^t^	97.21 ± 22.73	101.20 ± 27.46	**0**.011*
RNFL inferior^t^	130.99 ± 42.12	132.74 ± 51.73	**0**.034*
RNFL temporal^t^	72.11 ± 33.27	74.64 ± 26.53	**0**.046*
RNFL superior^t^	124.66 ± 34.62	126.18 ± 45.76	**0**.011*

Significant values are in [bold].

*p < 0.05, **p < 0.01, t: Paired Sample T Test, z: Mann Whitney U Test (Median, minimum and maximum values are given with mean and standard deviation).

There is no significant difference between the preoperative and postoperative results of patients without chiasmal distortion' SRCP fovea (*p* = 0.093), FAZ (*p* = 0.184), PVD whole (*p* = 0.185), PVD nasal (*p* = 0.118) and RNFL whole (*p* = 0.155) values. However, these patients' VF MD (*p* < 0.001), SRCP whole (*p* < 0.001), SRCP parafovea (*p* = 0.045), DRCP whole (*p* = 0.007), DRCP fovea (*p* = 0.006), DRCP parafovea (*p* = 0.040), PVD Peripapillary (*p* = 0.010), PVD inferior (*p* < 0.001), PVD temporal (*p* < 0.001), PVD superior (*p* < 0.001), RNFL nasal (*p* = 0.011), RNFL inferior (*p* = 0.034), RNFL temporal (*p* = 0.046) and RNFL superior (*p* = 0.011) values increased postoperatively.

The correlation between the visual recovery and the preoperative retinal parameters is shown in Table 4.

**Table 4:** Correlations between visual recovery and preoperative retinal parameters.

Preoperative parameters	Coefficient	Recovery	Preoperative parameters	Coefficient	Recovery
SRCP whole	r	− 0.487**	PVD nasal	r	− 0.071
	*p*	< **0**.**001**		*p*	0.410
SRCP fovea	r	0.056	PVD inferior	r	− 0.216*
	*p*	0.517		*p*	**0**.**011**
SRCP parafovea	r	− **0**.483**	PVD temporal	r	− 0.300**
	*p*	< **0**.**001**		*p*	< **0**.**001**
DRCP whole	r	− **0**.433**	PVD superior	r	− 0.121
	*p*	< **0**.**001**		*p*	0.161
DRCP fovea	r	− 0.052	RNFL whole	r	− 0.342**
	*p*	0.555		*p*	< **0**.**001**
DRCP parafovea	r	− 0.480**	RNFL nasal	r	− 0.082
	*p*	< **0**.**001**		*p*	0.343
FAZ	r	− 0.128	RNFL inferior	r	− .433**
	*p*	0.143		*p*	< **0**.**001**
PVD whole	r	0.068	RNFL temporal	r	− 0.204*
	*p*	0.431		*p*	**0**.**017**
PVD peripapillary	r	0.019	RNFL superior	r	− 0.278**
	*p*	0.826		*p*	**0**.**001**

Significant values are in [bold].

*p < 0.05. **p < 0.01, r: Correlations coefficient.

No significant correlation was found between visual recovery and SRCP fovea (r = 0.056 *p* = 0.517), DRCP fovea (r = − 0.052 *p* = 0.555), FAZ (r = − 0.128 *p* = 0.143), PVD (whole r = 0.068 *p* = 0.431, peripapillary r = 0.019 *p* = 0.826, nasal r = − 0.071 *p* = 0.410. superior r = − 0.121 *p* = 0.161) and RNFL nasal (r = − 0.082 *p* = 0.343) values.

There was a moderate negative correlation between visual recovery and SRCP (whole r = − .487 *p* < 0.001, parafovea r = − .483 *p* < 0.001), DRCP (whole r = − .433 *p* < 0.001, parafovea r = − .480 *p* < 0.001), PVD temporal r = − .300 *p* < 0.001, RNFL (whole r = − .342 *p* < 0.001, inferior r = − .433 *p* < 0.001), and a poor negative correlation between PVD inferior r = − .216 *p* = 0.011, RNFL (temporal r = − .204 *p* = 0.017, superior r = − .278 *p* = 0.001).

ROC analysis was performed to predict visual recovery with preoperative parameters. The area under the curve, sensitivity, and specificity results obtained from the ROC analysis for the prediction of visual recovery are shown in Table 5.

**Table 5:** ROC analyze of preoperative parameters for visual recovery prediction.

Test parameters	Area (AUC)	SD	*p*	Cut off	Sensitivity (%)	Specialty (%)	Margin (95)	
							Min	Max
SRCP whole	0.793	0.046	< 0.001	51.45	78.3	25	0.702	0.884
SRCP parafovea	0.820	0.038	< 0.001	53.95	68.5	20	0.746	0.894
DRCP whole	0.818	0.037	< 0.001	55.95	66.4	22.5	0.745	0.891
DRCP parafovea	0.820	0.039	< 0.001	59.05	62.0	15.0	0.744	0.896
PVD temporal	0.692	0.049	< 0.001	55.10	38.0	12.5	0.597	0.787
RNFL whole	0.690	0.055	0.001	119.5	27.2	12.5	0.582	0.799
RNFL inferior	0.712	0.062	< 0.001	144.75	51.1	25.0	0.591	0.833

Area under the curve (AUC): used to prediction of visual recovery.

**Figure 2:**
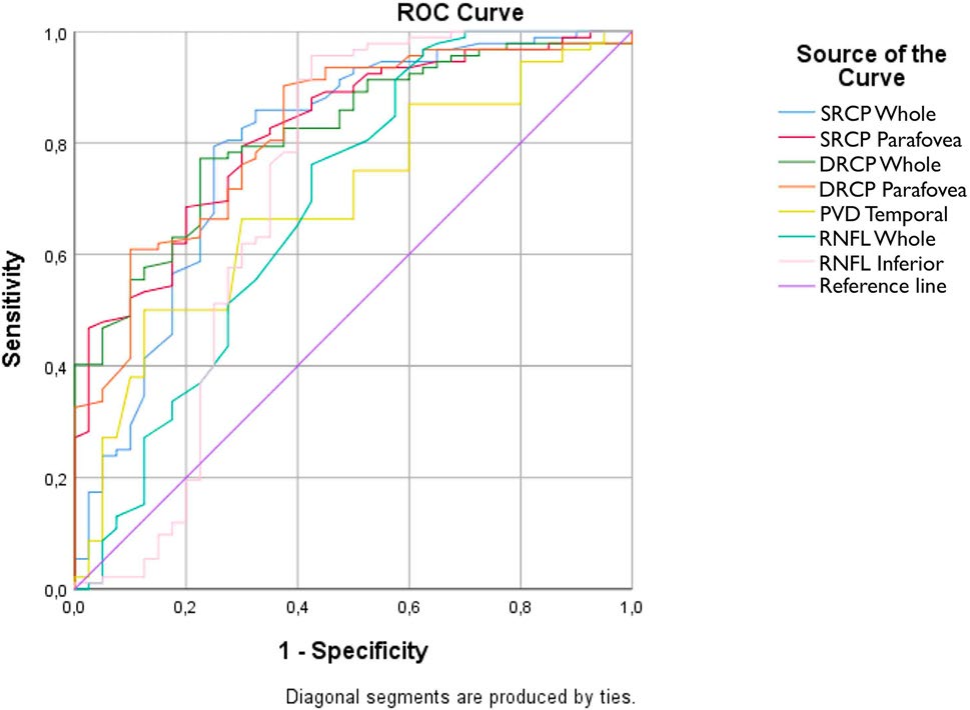
ROC curve of visual recovery prediction.

In this study, the size of the area under the ROC curve is statistically important in estimating the visual recovery. As a result of ROC analysis, the expected value of the area under the ROC curve is 0.50 when the eyes have no ability to distinguish between recovery and nonrecovery. In a perfect test, these values are expected to be 1.00. In the interpretation of the values under the curve; 0.90–1.00 = excellent, 0.80–0.90 = good, 0.70–0.80 = moderate, 0.60–0.70 = poor, and 0.50–0.60 = unsuccessful^20^. The ROC analysis result is shown in Fig. 2. In Table 5, statistically significant parameters SRCP (whole *p* < 0.001 cut off: 51.45, parafovea *p* < 0.001 cut off: 53.95), DRCP (whole *p* < 0.001 cut off: 55.95, parafovea *p* < 0.001 cut off: 59.05), PVD temporal *p* < 0.001 cut off: 55.10, RNFL (whole *p* = 0.001 cut off: 119.5, inferior *p* < 0.001 cut off: 144.75) is estimating the visual recovery.

## Discussion

In this study, we investigated the changes in retinal vascular density and retinal nerve layer thickness according to optic chiasm distortion in patients with SP tumor. We evaluated the patients before and after tumor removal and compared the results by distorted or undistorted optic chiasm. RNFL parameters were examined with OCT for evaluation of the optic disc. Foveal SRCP, DRCP, FAZ parameters, and Optic disc’s PVD parameters were examined with OCT-A. The relationship between vascular density data, RNFL thickness, and visual field testing was also investigated. We explored the predictive role of OCT-A in patients with no chiasmal distortion on MRI. We revealed visual recovery projection by investigating vascular modifications following the endoscopic endonasal surgery.

Preoperative retinal vascular densities were significantly decreased in Superficial Retinal Capillary Plexus (SRCP) (whole *p* < 0.001, fovea *p* = 0.025, parafovea *p* < 0.001), Deep Retinal Capillary Plexus (DRCP) (whole *p* < 0.001, fovea *p* = 0.003, parafovea *p* < 0.001), peripapillary Vascular Density (PVD) (whole *p* = 0.045, peripapillary *p* < 0.001, nasal *p* < 0.001, inferior *p* < 0.001, temporal *p* < 0.001), and Retinal Nerve Fiber Layer (RNFL)(nasal *p* = 0.024, inferior *p* < 0.001, temporal *p* < 0.001, superior *p* < 0.001) compared with healthy controls. The postoperative data of patients without chiasmal distortion compared with preoperative data. In postoperative evaluation, vascular densities of patients without chiasmal distortion were significantly increased in the SRCP (whole *p* < 0.001, parafovea *p* = 0.045), DRCP (whole *p* = 0.007, fovea *p* = 0.006, parafovea *p* = 0.040), PVD (peripapillary *p* = 0.010. inferior *p* < 0.001, temporal *p* < 0.001, superior *p* < 0.001), and RNFL (nasal *p* = 0.011, inferior, *p* = 0.034 temporal *p* = 0.046, superior *p* = 0.011). There were significant associations in the ROC analysis between the postoperative Visual Field Mean Deviation (VFMD) and SRCP (whole AUC = 0.793, *p* < 0.001, cut off = 51.45, parafovea AUC = 0.820. *p* < 0.001, cut off = 53.95), DRCP (whole AUC = 0.818, *p* < 0.001, cut off = 55.95, parafovea AUC = 0.820, *p* < 0.001, cut off = 59.05), PVD (temporal AUC = 0.692, *p* < 0.001, cut off = 55.10), and RNFL (whole AUC = 0.690, *p* = 0.001, cut off = 119.5, inferior AUC = 0.712, *p* < 0.001, cut off = 144.75) were found.

OCT, monitories, and measures RNFL and ganglion cell complex (GCC) structures in chiasmal distortion and optic neuropathy for many years^6,21,22^. OCT has a role in the early diagnosis and surgical timing of patients with SP tumors^22,23^. Also, postoperative visual recovery prediction with measurement of preoperative RNFL thickness may be possible in patients with chiasmal distortion^24–27^.

OCT-A allows a non-invasive examination of the retinal layers and provides quantifiable information of the vascular structure within the fovea and the optic disc^28–31^. Combining OCT and OCT-A data could provide deeper insights into the status of patients with SP tumor. This may provide a more accurate approach for the prognosis of optic neuropathy with chiasmal distortion^15^. PVD, SRCP, and DRCP parameters reduce in patients with optic neuropathy caused by chiasmal distortion. They compared with healthy controls, and there is a correlation with GCC, RNFL, and VF MD loss in patients with chiasmal distortion, suggesting that OCT-A could serve as a surrogate for retinal neural loss in compressive optic neuropathy and might be useful in its diagnosis and management^15,32–34^.

The factors associated with visual loss and factors related to VF MD recovery in patients with optic chiasm distortion are the degree of optic atrophy, the severity of VF defect, and the tumor size on MRI^35–39^.

It was thought the surgical timing and visual loss duration may also play a role in terms of visual improvement^40,41^ Hence, we considered the identified visual symptom duration and postoperative visual recovery may have any correlation with regard to postoperative VD MD, OCT, or OCT-A values but the statistical analysis was nonsignificant. Visual symptom duration is a subjective marker. It was difficult for the patient to notice the slowly developing bitemporal hemianopsia, which created bias. Wang et al. Mentioned a significant positive correlation between PVD and RNFL in patients with chiasmal distortion but the duration of symptoms did not appear to be associated with PVD and RNFL^35^.

Visual recovery progress in the first months after surgery and the improvement of visual field defects is a continuing process for at least 1 year^4,42,43^. Kerrison et al. showed progressive improvement of visual fields even more than 2 years after surgical decompression of the optic chiasm^4^.

Optic neuropathy-related visual field loss appeared when the optic chiasm distortion was above the reference line on the sagittal MRI image. Preoperative optic chiasm morphology is a predictive factor for visual outcome^34,44–46^. Optic chiasm distortion depending sagittal bending of the optic nerve is related to the deterioration of visual acuity in sellar and suprasellar lesions. Sagittal pituitary MRI is recommended for preoperative estimation for optic nerve bending^47^.

In this study, patients are grouped with MRI scans as chiasmal distortion or not. We compared the participants' preoperative retinal structural and vascular parameters and visual field tests. There was a significant difference between the groups in SRCP whole, SRCP parafovea, and PVD temporal parameters. Preoperative retinal parameters of the group with chiasmal distortion were significantly lower than the control group, except SRCP fovea and RNFL temporal parameters (Table 2). We demonstrated optic neuropathy, structural and vascular atrophy due to chiasmal distortion.

In the chiasmal distortion group, we could not find a correlation between the measurement of chiasmal distortion or tumor size and preoperative retinal values.

Patients without chiasmal distortion' preoperative SRCP whole, SRCP parafovea, DRCP whole, DRCP fovea, DRCP parafovea, RNFL temporal and RNFL superior parameters were found significantly lower than the control group. Although there was no chiasmal distortion on MRI, the presence of vessel atrophy in the foveal vascular parameters revealed that OCT-A has a value for the preoperative evaluation. The postoperative parameters of this group, SRCP (whole, parafovea), DRCP (whole, fovea, parafovea), PVD (peripapillary, inferior, temporal, superior), RNFL (nasal, inferior, temporal, superior), were significantly higher according to their preoperative results. (Table 3). We demonstrated that optic neuropathy and vascular atrophy improved in the postoperative period. The OCT-A usage was found to be sensitive and beneficial in the diagnosis of mild optic neuropathy and its follow-up.

Evaluation of a small number of eyes that did not improve functionally, we found that some preoperative retinal parameters were significant in predicting recovery. A correlation was found between postoperative VF MD and preoperative foveal vascular parameters SRCP (whole, parafovea), DRCP (whole, parafovea), PVD temporal, RNFL (whole, inferior, temporal, superior) parameters (Table 4). These findings revealed that the visual recovery potential increases with the increasing of SRCP (whole, parafovea), DRCP whole, parafovea, PVD (temporal, inferior), and RNFL (whole, inferior, temporal, and superior) results. However, a ROC analysis was performed for the parameters (Fig. 1). SRCP parafovea, and DRCP (whole, parafovea) foveal vascular density parameters were significant and we found that the cut-off values of these parameters can be used for the prediction of recovery.

In this study, SRCP parafovea (53.95), DRCP whole (55.95), and DRCP parafovea (59.05) results were good in estimating the potential of visual recovery, SRCP whole and RNFL inferior parameters had moderate estimation for the potential of visual recovery, PVD temporal and RNFL whole parameters had low effectiveness in the prediction of visual recovery.

We could not find a significant relationship between age, gender, or the secretory features of the tumor and functional improvement of the eyes.

To our knowledge, this study has one of the broadest sample sizes to reveal retinal vascular changes in a delicate way, particularly in patients without chiasmal distortion on MRI. Also, there is a potential to demonstrate OCT-A for postoperative expectations of visual recovery with one of the widest participants in the literature.

The limitation of the study is the inability long term results yet. Since the single-center study, some of our findings may not be applicable to studies with various patient groups. Further prospective and systematic OCT-A studies are needed to better understand the pathophysiology of microvascular changes in patients with SP tumor and long-term results for visual recovery.

Both endoscopic endonasal approach on SP tumor patients and OCTA examination in ophthalmology widely used in many clinics. A multi-center study could be design and comprehensive results can be achieved.

A study can be designed with a group of patients containing a similar number of patients with SP tumor, and results can be obtained at postoperative 6 months, 1-year anniversary, and 2-year anniversary by repeating tests in follow-up appointments. This way, long-term OCTA results can be illuminated.

## Conclusion

We investigated preoperative and postoperative retinal microcirculation alterations with OCT-A for eyes with chiasmal distortion and non-chiasmal distortion. Patient’s eyes with non-chiasmal distortion were found to be associated with optic neuropathy and vascular atrophy. In the postoperative period, functional and structural recovery has been revealed in these eyes. We demonstrated the recovery of mild retinal vascular atrophy in patients without chiasmal distortion.

We researched the prognostic value of visual recovery potential with OCT-A. Our findings suggest a potential role of preoperative OCT-A measurements in the assessment of postoperative visual recovery. Further studies are required to confirm these results and reveal the advantage of hardened OCT-A practice in clinical routine.

## Data Availability

The datasets generated during and/or analyzed during the current study (statistical analysis plan) are available from the corresponding author on reasonable request.
